# Trends in the Use of Oral Anticoagulants for Adults With Venous Thromboembolism in the US, 2010-2020

**DOI:** 10.1001/jamanetworkopen.2023.4059

**Published:** 2023-03-22

**Authors:** Geetha S. Iyer, Helen Tesfaye, Nazleen F. Khan, Heidi Zakoul, Katsiaryna Bykov

**Affiliations:** 1 Division of Pharmacoepidemiology and Pharmacoeconomics, Department of Medicine, Brigham and Women’s Hospital and Harvard Medical School, Boston, Massachusetts; 2 Department of Epidemiology, Harvard T.H. Chan School of Public Health, Boston, Massachusetts

## Abstract

**Importance:**

The introduction of direct oral anticoagulants (DOACs) has transformed the treatment of venous thromboembolism (VTE). Large health care databases offer valuable insight into how oral anticoagulants (OACs) are used in clinical practice and may aid in understanding reasons for changes in therapy.

**Objectives:**

To evaluate prescribing patterns of OACs for patients with VTE and identify clinical events that precede treatment changes.

**Design, Setting, and Participants:**

This retrospective cohort study used data from a public (Medicare fee-for-service) and a commercial (IBM MarketScan) health insurance database on 298 609 patients initiating OACs within 90 days of index VTE hospitalization from January 1, 2009, to December 31, 2020. Statistical analysis was conducted from April to August 2022.

**Exposures:**

Warfarin and the DOACs rivaroxaban, apixaban, dabigatran, and edoxaban.

**Main Outcomes and Measures:**

Characteristics of patients initiating different OACs, along with trends over time of patients initiating OACs, were compared. Time receiving continuous anticoagulant therapy, patterns of anticoagulant discontinuation (treatment gap of ≥30 days), and treatment switches were assessed. Clinical events in the 30 days preceding treatment modifications were identified.

**Results:**

A total of 203 378 individuals with Medicare (mean [SD] age, 76.9 [7.6] years; 122 554 women [60.3%]) and 95 231 with commercial insurance (mean [SD] age, 57.6 [15.8] years; 47 139 women [49.5%]) were included (N = 298 609). Warfarin was the most frequent OAC prescribed (163 044 [54.6%]), followed by rivaroxaban (66 882 [22.3%]) and apixaban (65 997 [22.1%]). The proportion of patients initiating DOACs increased from 0% in 2010 to 86.8% (22 420 of 25 817) in 2019 for patients with Medicare and 92.1% (4012 of 4357) in 2020 for commercially insured patients. Patients with chronic kidney disease were more likely to initiate warfarin (35 561 [11.9%]) or apixaban (16 294 [5.5%]) than rivaroxaban (10 136 [3.4%]), and those with a history of bleeding were more likely to initiate apixaban (5424 [1.8%]) than rivaroxaban (3007 [1.0%]). Overall, patients received persistent OAC treatment for approximately 6 months (Medicare: median, 175 days [IQR, 76-327 days]; commercial insurance: median, 168 days [IQR, 83-279 days]). A total of 33 011 patients (11.1%) switched anticoagulant therapy within a year. Switching to another anticoagulant was preceded most frequently by codes for a VTE diagnostic procedure (27.2% of all switchers [8983 of 33 011]).

**Conclusions and Relevance:**

This cohort study using data from 2 US health insurance databases suggests that most patients with VTE continued oral anticoagulant treatment for approximately 6 months. Clinical reasons for modifying anticoagulant therapy were identified in one-third of patients. Identifying reasons for treatment modification is crucial for generating valid evidence on drug safety and effectiveness.

## Key Points

**Question:**

What are the use patterns of oral anticoagulants (OACs) and the clinical reasons for treatment modification among patients with venous thromboembolism (VTE) from 2010 to 2020?

**Findings:**

In this cohort study of 298 609 patients with VTE from 2 large health care databases in the US, warfarin (55%) was the most frequent OAC prescribed, followed by rivaroxaban (22%) and apixaban (22%); most patients continued their treatment for approximately 6 months. Potential clinical reasons for modifying anticoagulant therapy were identified using insurance claims from the prior 30 days for one-third of patients.

**Meaning:**

With the changing landscape of VTE management after the introduction of direct oral anticoagulants, health care databases can be used to better understand treatment decisions and modifications and aid in designing comparative effectiveness and safety studies for oral anticoagulants.

## Teaser

This cohort study uses data from Medicare fee-for-service and a commercial insurance database to evaluate prescribing patterns of oral anticoagulants for patients with venous thromboembolism and identify clinical events that precede treatment changes.

## Introduction

Venous thromboembolism (VTE), comprising both deep vein thrombosis (DVT) and pulmonary embolism (PE), was categorized by the US Surgeon General as a major public health problem in 2008.^1^ This condition affects approximately 1 to 2 per 1000 people in the US annually, with the incidence increasing markedly with age.^2^ Approximately 28% of individuals die within 30 days of the VTE episode and 36% of individuals die within 1 year of the VTE episode, with PE having a much higher incidence than DVT.^3^ The mainstay of treatment for the prevention of VTE recurrence is anticoagulation, with either oral or parenteral agents.

Although warfarin was the main oral anticoagulant used for decades in patients with VTE, several direct oral anticoagulants (DOACs) were approved by the US Food and Drug Administration (FDA) for secondary prevention of VTE in the 2010s (rivaroxaban in 2012, dabigatran and apixaban in 2014, and edoxaban in 2015).^4,5,6,7,8^ A few studies evaluating the prescribing patterns of oral anticoagulants (OACs) found a rapid uptake of these newer agents, especially rivaroxaban and apixaban, among patients in the US,^9,10,11,12^ attributed to DOACs’ lower bleeding risk, convenient dosing schedules, reduced need for laboratory test monitoring, and comparable or superior efficacy to warfarin. Most of these studies have been conducted among patients with nonvalvular atrial fibrillation or patients with any indication. However, not all patients prescribed OACs for secondary prevention of VTE are recommended to receive lifelong anticoagulation. The 2016 guidelines proposed by the American College of Chest Physicians (ACCP)^13^ and the 2020 guidelines proposed by the American Society of Hematology (ASH),^14^ recommend that the duration of anticoagulation depend on whether the initial VTE event was provoked by the presence of certain clinical risk factors or was idiopathic,^15^ as well as dependent on patients’ risk of bleeding.

Our study aimed to understand the use patterns of OACs prescribed for the secondary prevention of VTE in routine clinical practice. Specifically, we focused on describing the characteristics of patients with VTE initiating different OACs (warfarin and DOACs), characterizing the trends over time of OAC initiation among patients with VTE, and assessing adherence and persistence to OAC therapy, with evaluation of patterns and reasons for treatment modification such as discontinuation and switching between different OACs.

## Methods

### Data Source

We used 2 US-based health care claims databases, covering both publicly and commercially insured individuals (ie, Medicare fee-for-service^16^ and IBM MarketScan). These databases contain comprehensive longitudinal deidentified information on enrollment, demographic characteristics, and inpatient and outpatient billed diagnoses and procedures, as well as pharmacy claims for filled prescription medications. The diagnoses and procedures were identified using *International Classification of Diseases, Ninth Revision* codes and *International Statistical Classification of Diseases and Related Health Problems, Tenth Revision* codes, the Healthcare Common Procedure Coding System, and the *Current Procedural Terminology*. Information on medications was identified using National Drug Codes. The study was approved by the Mass General Brigham institutional review board, which granted a waiver of informed consent as the data were deidentified. The study followed the Strengthening the Reporting of Observational Studies in Epidemiology (STROBE) reporting guideline.

### Study Design and Population

We identified individuals aged 18 years or older between January 1, 2010, and December 31, 2020, from IBM MarketScan and individuals aged 66 years or older between January 1, 2010, and December 31, 2019, from Medicare who initiated an OAC (warfarin, dabigatran, rivaroxaban, apixaban, or edoxaban) within 90 days after discharge from VTE hospitalization. The index VTE event was identified using hospital discharge diagnoses codes for either PE or DVT in the primary position (eTable in Supplement 1). Patients were required to have at least 365 days of medical and pharmacy insurance enrollment prior to the index VTE event, with no hospitalization for VTE or anticoagulant prescription filled during that time. If patients had more than 1 VTE hospitalization fulfilling the criteria, the first hospitalization was considered the index VTE hospitalization. The date of the initial OAC prescription was considered the index prescription date. Patients were followed up from the index prescription date until the loss of their medical or pharmacy insurance eligibility, death, end of the study period (365 days), or end of data availability.

### Demographic and Clinical Characteristics

We assessed the demographic and clinical characteristics of the included patients (Table 1). Demographic characteristics were assessed on the index prescription date and included age, sex, and race and ethnicity (as reported in the Medicare database based on data from the Social Security Administration and the results of an imputation algorithm applied to the source data; race and ethnicity were not available in the IBM MarketScan database).^17^ Clinical characteristics were assessed over the time from 365 days before the index VTE event admission date to the index VTE event discharge date and included characteristics of the index VTE hospitalization, such as type of VTE, lifestyle and comorbid conditions, prescription medications, and measures of health care use. The complete list of characteristics is included in Table 1.

**Table 1.** Demographic and Clinical Characteristics of Patients With VTE Initiating OACs From 2 US Health Care Claims Databases, 2010-2020^a^

Characteristic	Patients, No. (%)					
	Medicare (n = 203 378)^b^			IBM MarketScan (n = 95 231)^b^		
	Warfarin (n = 101 765)	Rivaroxaban (n = 47 827)	Apixaban (n = 51 691)	Warfarin (n = 61 279)	Rivaroxaban (n = 19 055)	Apixaban (n = 14 306)
Demographic characteristics						
Age, mean (SD), y	77.2 (7.7)	75.8 (7.2)	77.2 (7.8)	58.5 (16.0)	55.1 (15.2)	57.1 (15.3)
Women	62 910 (61.8)	27 568 (57.6)	30 879 (59.7)	30 383 (49.6)	9265 (48.6)	7201 (50.3)
Race and ethnicity^c^						
Black	12 489 (12.3)	4875 (10.2)	5905 (11.4)	NA	NA	NA
White	85 132 (83.7)	40 849 (85.4)	43 431 (84.0)			
Other^d^	4144 (4.0)	2103 (4.4)	2355 (4.6)			
Initial VTE episode and OAC prescription fill						
Type of VTE						
DVT	43 288 (42.5)	15 197 (31.8)	15 895 (30.7)	24 063 (39.3)	5854 (30.7)	4112 (28.7)
PE	58 477 (57.5)	32 630 (68.2)	35796 (69.3)	37 216 (60.7)	13 201 (69.3)	10 194 (71.3)
Clinical risk factors						
Provoked VTE						
Transient	4664 (4.6)	1867 (3.9)	2204 (4.37)	1897 (3.1)	478 (2.5)	453 (3.2)
Persistent	26 811 (26.4)	13 730 (28.7)	14 527 (28.1)	9769 (15.9)	2933 (15.4)	2361 (16.5)
Unprovoked VTE	70 290 (69.1)	32 230 (67.4)	34 960 (67.6)	49 613 (81.0)	15 644 (82.1)	11 492 (80.3)
Duration of initial hospitalization, median (IQR), d	5 (4-7)	4 (3-5)	4 (3-5)	5 (3-7)	3 (2-5)	3 (3-5)
Time to initiate OAC from discharge, median (IQR), d	0 (0-1)	0 (0-13)	1 (0-20)	0 (0-1)	0 (0-10)	1 (0-22)
Use of parenteral anticoagulant before initiating OAC	34 450 (33.9)	2013 (4.2)	1581 (3.1)	27 697 (45.2)	894 (4.7)	512 (3.6)
Comorbidities						
Smoking	38 239 (37.6)	21 394 (44.7)	23 853 (46.2)	9257 (15.1)	3701 (19.4)	2983 (20.9)
Obesity	21 211 (20.8)	13 418 (28.1)	17 074 (33.0)	8799 (14.4)	5013 (26.3)	5088 (35.6)
Hypertension	85 182 (83.7)	40 048 (83.7)	46 191 (89.4)	34 115 (55.7)	10 297 (54.0)	9207 (64.4)
Type 2 diabetes	39 373 (38.7)	16 506 (34.5)	18 922 (36.6)	13 438 (21.9)	3841 (20.2)	3254 (22.8)
Hyperlipidemia	70 770 (75.9)	26 665 (76.5)	16 429 (77.0)	25 613 (41.8)	7955 (41.8)	6653 (46.5)
Anemia	46 937 (46.1)	18 269 (38.2)	22 134 (42.8)	13 651 (22.3)	4064 (21.3)	3704 (25.9)
Atrial fibrillation	11 480 (11.3)	4415 (9.2)	5435 (10.5)	1909 (3.1)	505 (2.7)	432 (3.0)
Ischemic						
Stroke	17 492 (17.2)	7074 (14.8)	8418 (16.3)	3986 (6.5)	961 (5.0)	855 (6.0)
Heart disease	39 112 (38.4)	18 057 (37.8)	22 904 (44.3)	9535 (15.6)	3012 (15.8)	2956 (20.7)
Congestive heart disease	19 191 (18.9)	6964 (14.6)	10 282 (19.9)	5047 (8.2)	1354 (7.1)	1480 (10.4)
Peripheral artery disease	22 584 (22.2)	8627 (18.0)	10 164 (19.7)	4100 (6.7)	1003 (5.3)	845 (5.9)
Coagulation defects	3813 (3.8)	1321 (2.8)	1411 (2.7)	2183 (3.6)	539 (2.8)	369 (2.6)
History of bleeding events	3301 (3.2)	2265 (4.7)	4289 (8.3)	625 (1.0)	742 (3.9)	1135 (7.9)
Abnormal liver function	12 308 (12.1)	6647 (13.9)	7460 (14.4)	5219 (8.5)	1961 (10.3)	1742 (12.2)
Acute kidney disease	19 120 (18.8)	7038 (14.7)	11 241 (21.8)	5196 (8.5)	1429 (7.5)	1656 (11.6)
Chronic kidney disease, stage IV or higher	29 878 (29.4)	9073 (19.0)	14 854 (28.7)	5683 (9.3)	1063 (5.6)	1440 (10.1)
Peptic ulcer disease	47 905 (47.1)	22 041 (46.1)	24 875 (48.1)	13 983 (22.8)	4575 (24.0)	3956 (27.7)
Prescription medication use						
Statins	51 754 (50.9)	24 195 (50.6)	26 952 (52.1)	19 117 (31.20)	5070 (26.6)	4191 (19.3)
Antiplatelets	13 150 (12.9)	4850 (10.1)	5844 (11.3)	3913 (6.4)	944 (5.0)	897 (6.3)
β-Blockers	48 160 (47.3)	19 718 (41.2)	23 266 (45.0)	16 636 (27.2)	4113 (21.6)	3546 (24.8)
ACE inhibitors	35 498 (34.9)	14 726 (30.8)	15 480 (30.0)	14 597 (23.8)	3823 (20.1)	3000 (21.0)
Angiotensin receptor blockers	21 738 (21.4)	11 040 (23.1)	12 820 (24.8)	9150 (14.9)	2679 (14.1)	2285 (16.0)
Antidiabetics	23 901 (23.5)	9729 (20.3)	11 576 (22.4)	9609 (15.7)	2640 (13.9)	2273 (15.9)
Estrogen (alone or with progestins)	2013 (2.0)	1129 (2.4)	1185 (2.3)	6472 (10.6)	2465 (12.9)	1761 (12.3)
NSAIDs	26 607 (26.2)	13 829 (28.9)	14 472 (28.0)	17 652 (28.8)	6083 (31.9)	4522 (31.6)
Measures of health care use						
Influenza vaccine	63 119 (62.0)	30 923 (64.7)	34 601 (66.9)	13 956 (22.8)	5133 (26.9)	4267 (29.8)
No. of distinct medications filled, median (IQR)	11 (7-16)	10 (6-15)	11 (7-16)	8 (4-13)	8 (4-13)	8 (4-13)
No. of hospitalizations, median (IQR)	1 (1-2)	1 (1-2)	1 (1-2)	1 (1-2)	1 (1 - 2)	1 (1-2)
No. of emergency department visits, (median and IQR)	4 (2-7)	4 (2-7)	4 (3-8)	3 (2-5)	3 (2-5)	3 (2-6)
No. of ambulatory care visits, median (IQR)	36 (20-60)	34 (19-58)	36 (20-60)	20 (9-37)	18 (8-35)	19 (9-37)

Abbreviations: ACE, angiotensin-converting enzyme; DVT, deep vein thrombosis; NA, not available; NSAIDs, nonsteroidal anti-inflammatory drugs; OAC, oral anticoagulant; PE, pulmonary embolism; VTE, venous thromboembolism.

^a^ Medicare database, 2010-2019, and IBM MarketScan database, 2010-2020.

^b^ Medicare: 2025 patients initiated dabigatran, 70 patients initiated edoxaban; IBM MarketScan: 561 patients initiated dabigatran, 29 patients initiated edoxaban.

^c^ Race and ethnicity data were only available from the Medicare database.

^d^ Other included those belonging to Asian, Hispanic, Native American or Alaska Native, and unknown racial and ethnic groups. Only 1.6% of patients are in the unknown category.

The index VTE event was defined as provoked by major transient risk factors if the patient had an inpatient code for major trauma, fracture, or major surgery or was hospitalized for 3 days or more in the 90 days before the index VTE admission.^15,18,19^ Persistent provoked VTE was defined as the presence of a cancer diagnosis code on 2 distinct dates in the 365 days before the index VTE admission. The remaining VTE events were classified as unprovoked. We also identified the time from the index VTE event discharge date to the first OAC prescription fill and whether individuals filled a prescription of parenteral anticoagulants before initiating an OAC.

### Patterns of OAC Initiation and Use

We used pharmacy prescription claims to assess the patterns of OAC use among patients with VTE, with the focus on the following: initiating treatment with OACs, duration of therapy, and patterns of switching and discontinuation. We identified the first OAC prescription filled after the index VTE hospitalization and evaluated how the trends in the initiated OAC changed over time. We had access to only a 5% random sample of Medicare patients initiating warfarin from 2010 to 2011 and all patients initiating warfarin from 2012 to 2019.

The main measure was the cumulative time receiving OACs before discontinuing treatment (ie, the duration of therapy [median and IQR, in days]). We defined patients who discontinued treatment as those without an OAC prescription fill within 30 days after the end of days’ supply from a previous OAC fill. The date of discontinuation was the day after the end of days’ supply from the previous OAC fill. We calculated the proportion of patients with a single prescription fill (ie, with no OAC prescription fill after their initial fill during their follow-up). We calculated the measures among the entire study population, as well as among subgroups stratified by the type of VTE (DVT or PE), whether the initial VTE episode was provoked, and recent history of bleeding, defined as any diagnosis code for bleeding during the year before the index VTE event.

### Clinical Events Preceding OAC Discontinuation and Switching

We identified patients who modified their therapy (ie, those who discontinued OAC) or switched anticoagulants, defined as filling a prescription for another anticoagulant before discontinuation of the initial OAC (from warfarin to DOAC, from DOAC to warfarin, or from DOAC to DOAC). The date of switching was the date of the prescription fill of a different OAC than the initial OAC dispensed. For these patients, we sought to detect events that may have triggered these modifications by identifying prespecified clinical events in the 30 days before the date of discontinuation or switching.

The prespecified clinical events preceding discontinuation included (1) any health care encounter with a code for bleeding, including gastrointestinal, intracranial, or other sources of bleeding; (2) contraindications for OAC therapy, including acute kidney failure and stage IV or worse chronic kidney disease (including dialysis); and (3) any hospitalization. The clinical events preceding switching included (1) any diagnostic procedures for VTE (eg, compression ultrasonography, ventilation-perfusion scan, or chest computed tomography scan); (2) any health care encounter with a code for VTE, atrial fibrillation, or ischemic stroke; (3) a code for cancer; (4) a code for bleeding; and (5) any hospitalization.

### Statistical Analysis

Statistical analysis was conducted from April to August 2022. We compared the baseline characteristics of patients based on the OAC initiated. Continuous variables are reported as mean (SD) or median (IQR) values, and categorical variables are reported as percentages. To illustrate the uptake of DOACs over time, we plotted the proportion of patients initiating each of the 5 OACs against time in 3-month increments. All analyses were conducted using SAS, version 9.4 (SAS Institute Inc).

## Results

A total of 203 378 patients with Medicare (mean [SD] age, 76.9 [7.6] years; 122 554 women [60.3%]) and 95 231 with commercial insurance (mean [SD] age, 57.6 [15.8] years; 47 139 women [49.5%]) initiated an OAC after discharge from VTE hospitalization (N = 298 609) (Table 1). Among Medicare patients initiating OACs, 101 765 (50.0%) initiated warfarin, 51 691 (25.4%) initiated apixaban, 47 827 (23.5%) initiated rivaroxaban, 2025 (1.0%) initiated dabigatran, and 70 (0.03%) initiated edoxaban. Among the commercially insured patients initiating OACs, 61 279 (64.3%) initiated warfarin, 19 055 (20.0%) initiated rivaroxaban, 14 306 (15.0%) initiated apixaban, 562 (0.6%) initiated dabigatran, and 29 (0.03%) initiated edoxaban.

Table 1 shows the demographic and clinical characteristics of the study population. As expected, Medicare beneficiaries were older and had more comorbidities compared with those with commercial insurance. In both databases, PE comprised the majority of index VTE events. Medicare patients initiating OACs had a lower proportion of VTE events categorized as unprovoked by either transient or persistent clinical risk factors (68.3% [138 831 of 203 378]) compared with commercially insured patients (81.1% [77 229 of 95 231]). Patients with obesity and those with smoking history, both important risk factors for VTE, were more likely to initiate a DOAC rather than warfarin. Patients with chronic kidney disease were more likely to initiate warfarin (35 561 of 298 609 [11.9%]) or apixaban (16 294 of 298 609 [5.5%]) compared with rivaroxaban (10 136 of 298 609 [3.4%]), and those with a history of bleeding were more than twice as likely to initiate apixaban (5424 of 298 609 [1.8%]) compared with rivaroxaban (3007 of 298 609 [1.0%]).

### Patterns of OAC Initiation Over Time

The proportion of patients initiating DOACs increased from 0% in 2010 to 86.8% (22 420 of 25 817) in 2019 for patients with Medicare and 92.1% (4012 of 4357) in 2020 for commercially insured patients. The Figure shows the trends in OAC initiation over the study period in the 2 databases. In Figure, A, the timeline starts from 2012 as we had access to only a 5% random sample of patients with Medicare who were receiving warfarin until 2012. In Figure, A and B, patients almost exclusively initiated warfarin until the last quarter of 2012. After rivaroxaban was approved by the FDA for the secondary prevention of VTE in late 2012, it rapidly gained market share, and 31.9% of patients (2897 of 9070) in both databases combined were initiating rivaroxaban in the last quarter of 2014. However, with the FDA’s approval of apixaban in 2014, the proportion of patients initiating rivaroxaban plateaued and then decreased by the end of the study period. Apixaban had a similar rapid uptake after its approval with 34.9% of patients (2918 of 8357) initiating it in the last quarter of 2016, and 68.0% (5257 of 7734) patients initiating it by end of study in both databases combined. Only 10.8% of patients (836 of 7734) initiated warfarin by end of study period in both databases combined.

**Figure.**
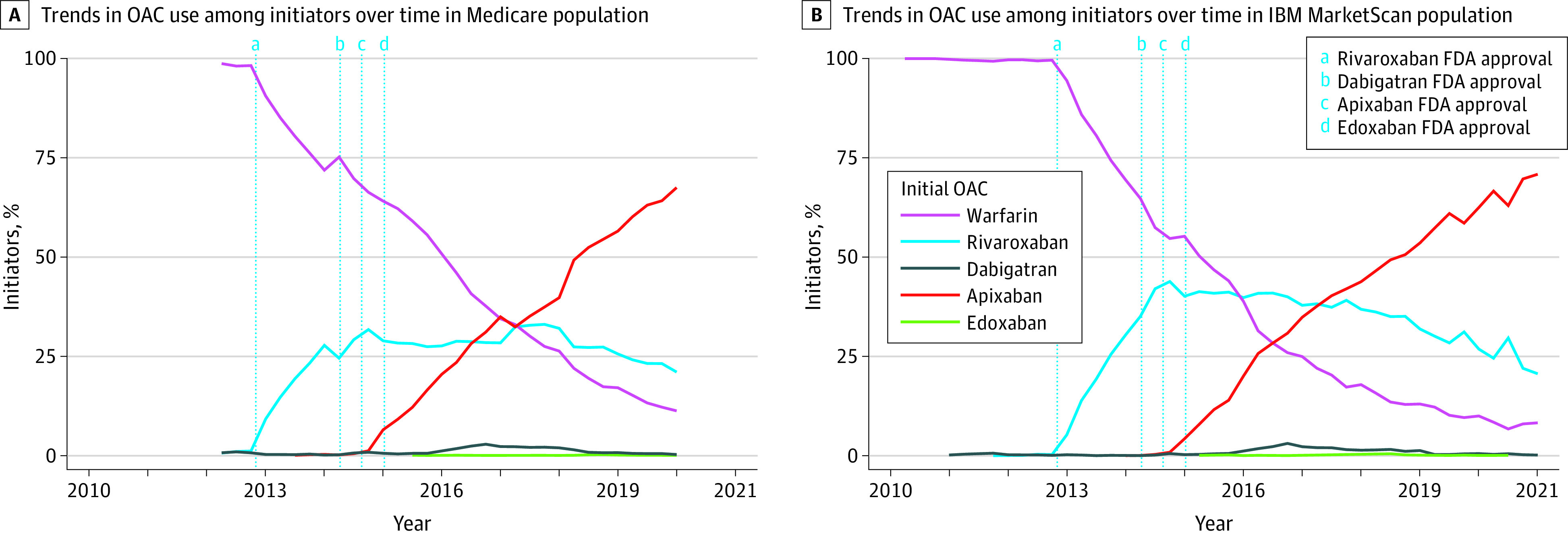
Trends in Oral Anticoagulants (OACs) Initiated Over Time (2010-2020) Among Patients With Venous Thromboembolism From 2 US Health Care Claims Databases A, Medicare population (2012-2019). B, IBM MarketScan population (2010-2020). The vertical dotted lines denote the time of US Food and Drug Administration (FDA) approval of the newer direct oral anticoagulants for secondary prevention of venous thromboembolism.

### Measures of Use

Table 2 presents the measures of OAC use in our study population. In both databases, we observed that patients were persistent with the treatment for approximately 6 months (Medicare: median, 175 days [IQR, 76-327 days]; commercial insurance: median, 168 days [IQR, 83-279 days]). Patients with PE as their index VTE event were persistent for approximately 20 days longer than the patients with DVT as their index VTE event. Those with a history of bleeding had a shorter time taking OACs (Medicare: median, 121 days [IQR, 46-234 days]; commercial insurance: median, 133 days [61-223 days]) compared with the overall population.

**Table 2.** Measures of Use Among Patients With Venous Thromboembolism Initiating OACs From 2 US Health Care Claims Databases, 2010-2020^a^

Characteristic	Medicare (n = 203 378)		IBM MarketScan (n = 95 231)	
	Single dispensing, No. (%)	Time taking OACs, median (IQR), d	Single dispensing, No. (%)	Time taking OACs, median (IQR), d
**Overall**	**20 385 (10.2)**	**175 (76-327)**	**7405 (7.8)**	**168 (83-279)**
Risk factors				
Provoked				
Transient	750 (8.5)	181 (90-311)	209 (7.3)	155 (76-219)
Persistent	5877 (10.5)	167 (69-331)	1897 (12.5)	138 (56-252)
Unprovoked	13 758 (9.9)	172 (76-326)	5302 (6.9)	171 (86-288)
Type of index event				
Pulmonary embolism	12 076 (9.4)	179 (81-340)	4511 (7.4)	171 (82-281)
Deep vein thrombosis	8307 (11.1)	159 (68-307)	2894 (8.5)	158 (76-272)
History of bleeding	1730 (17.2)	121 (46-234)	308 (12.1)	133 (61-223)

Abbreviation: OAC, oral anticoagulant.

^a^ Medicare database, 2010-2019, and IBM MarketScan database, 2010-2020.

A total of 20 385 patients with Medicare (10.2%) and 7405 patients with commercial insurance (7.8%) filled only the initial prescription of OACs over the follow-up time, most of which were for a 30-day supply. Those with a history of bleeding were more likely to fill only 1 prescription compared with the overall population (17.2% [n = 1730] of patients with Medicare and 12.1% [n = 308] of patients with commercial insurance).

### Clinical Events Preceding Discontinuation and/or Switching

Of the 203 378 patients with Medicare, 51 455 (25.3%) discontinued treatment and 26 439 (13.0%) switched to a different OAC. Of the 95 231 patients with commercial insurance, 21 236 (22.3%) discontinued treatment and 6572 (6.9%) switched to a different OAC.

Of those who discontinued, we identified a clinical event in the 30 days preceding discontinuation for 11 062 of 51 455 patients with Medicare (21.5%) and 2612 of 21 236 patients with commercial insurance (12.3%) (Table 3). The most common clinical event was inpatient hospitalization (6998 of 51 455 patients with Medicare [13.6%] and 1636 of 21 236 patients with commercial insurance [7.7%]). We also observed that 3860 of 51 455 patients with Medicare (7.5%) and 956 of 21 236 patients with commercial insurance (4.5%) had a health care encounter for bleeding in the 30 days preceding discontinuation.

**Table 3.** Clinical Events Preceding Discontinuation of Oral Anticoagulant Therapy Among Patients With Venous Thromboembolism From 2 US Health Care Claims Databases, 2010-2020^a^

Clinical event	Patients, No. (%)	
	Medicare (n = 51 455)	IBM MarketScan (n = 21 236)
Any event	11 062 (21.5)	2612 (12.3)
Bleeding complications		
Any	3860 (7.5)	956 (4.5)
Gastrointestinal	719 (1.4)	106 (0.5)
Intracranial	154 (0.3)	43 (0.2)
Other	2830 (5.5)	660 (3.1)
Absolute or relative contraindications		
Acute kidney failure	1798 (3.5)	275 (1.3)
Chronic kidney disease	3705 (7.2)	489 (2.3)
Hospitalization, any	6998 (13.6)	1636 (7.7)

^a^ Medicare database, 2010-2019, and IBM MarketScan database, 2010-2020.

Among the patients who switched OAC therapy (33 011 [11.1%]; Table 4), most switched from one of the DOACs to warfarin (13 873 of 26 439 patients with Medicare [52.5%] and 4182 of 6572 patients with commercial insurance [63.6%]), followed by warfarin to DOACs (5337 of 26 439 patients with Medicare [20.2%] and 991 of 6572 patients with commercial insurance [15.1%]). We were able to identify a clinical event in the 30 days preceding switching in close to half of all patients who switched. The most common clinical events were health care encounters with a VTE diagnostic procedure (8983 of 33 011 [27.2%]), health care encounters with a code for VTE (7126 of 33 011 [21.6%]), and hospitalization (6680 of 33 011 [20.2%]). Those who switched from a warfarin to a DOAC were more likely to have a code for a VTE diagnostic procedure, a health care encounter for VTE, or a code for bleeding in the 30 days preceding switching compared with those who switched from DOAC to warfarin.

**Table 4.** Clinical Events Preceding Switching of Oral Anticoagulant Therapy Among Patients With VTE From 2 US Health Care Claims Databases, 2010-2020^a^

Clinical event	Patients, No. (%)					
	Medicare (n = 26 439)			IBM MarketScan (n = 6572)		
	Warfarin to DOAC (n = 5337)	DOAC to Warfarin (n = 13 873)	DOAC to DOAC (n = 7229)	Warfarin to DOAC (n = 991)	DOAC to Warfarin (n = 4182)	DOAC to DOAC (n = 1399)
Any event^b^	3405 (63.8)	6451 (46.5)	3296 (45.6)	696 (70.2)	1627 (38.9)	659 (47.1)
VTE diagnostic procedures						
Compression ultrasound or ventilation perfusion scan or chest CT scan	1767 (33.1)	3578 (25.8)	1800 (24.9)	417 (42.1)	954 (22.8)	467 (33.4)
Thromboembolic events						
VTE	1755 (32.9)	2594 (18.7)	1388 (19.2)	373 (37.6)	745 (17.8)	271 (19.4)
Atrial fibrillation	267 (5.0)	609 (4.4)	347 (4.8)	19 (1.9)	67 (1.6)	18 (1.3)
Ischemic stroke	112 (2.1)	125 (0.9)	87 (1.2)	17 (1.7)	21 (0.5)	11 (0.8)
Bleeding complications	507 (9.5)	291 (2.1)	159 (2.2)	71 (7.2)	50 (1.2)	18 (1.3)
Cancer	1003 (18.8)	2386 (17.2)	1266 (17.5)	117 (11.8)	456 (10.9)	142 (10.1)
Hospitalization, any	1772 (33.2)	2704 (19.5)	1222 (16.9)	345 (34.8)	464 (11.1)	173 (12.3)

Abbreviations: CT, computed tomography; DOAC, direct oral anticoagulant; VTE, venous thromboembolism.

^a^ Medicare database, 2010-2019, and IBM MarketScan database, 2010-2020.

^b^ Any of the events listed.

## Discussion

The results from our study describe use patterns of OACs among patients with VTE from two large health insurance databases representative of the US population. We observed that the uptake of newer DOACs, such as rivaroxaban and apixaban, rapidly increased after their FDA approval in early 2010s, with patients initiating warfarin comprising only 10% to 15% of all patients initiating OACs by end of the study. By 2020, apixaban became the most commonly initiated OAC among patients with VTE. This preference of DOACs vs warfarin has been noted previously in studies globally, mostly conducted among patients with nonvalvular atrial fibrillation.^10,20,21,22^ However, in contrast to studies of patients with nonvalvular atrial fibrillation, we observed very few patients who filled a prescription for dabigatran as their initial OAC.

According to the 2016 ACCP guidelines,^13^ patients with VTE were to be treated with OACs for a minimum of 3 months to prevent any VTE recurrence, with the newer DOACs preferred over warfarin. Treatment for 3 months was recommended for transient provoked VTE and for patients with a high risk of bleeding. Extended therapy was recommended for those with persistent provoked VTE and for those with unprovoked VTE, with treatment duration conditional on bleeding risk and risk of VTE recurrence. However, in the most recent guidelines (ie, the 2020 ASH guidelines for VTE treatment^14^ and 2021 ACCP guidelines^23^), patients with unprovoked VTE are also recommended to receive extended therapy beyond 3 months. In our study, the median duration of OAC therapy overall was approximately 5 to 6 months. However, we did not observe much variability in median duration based on whether the index VTE event was provoked or unprovoked. Patients with a history of bleeding had a lower median duration of therapy (approximately 4 months), which was in line with the recommendations. These results were similar to those observed by Martinez et al^24^ in 2018.

When evaluating treatment duration, it is also important to consider clinical events that may lead to treatment change. One of the aims of the study was to identify clinical reasons for therapy discontinuation or switching to a different OAC. As large health care databases are increasingly favored for studies on comparative safety and effectiveness of drugs,^25,26^ understanding the reasons for treatment decisions is paramount because it allows for better bias control. In our study, we identified a prespecified clinical event preceding discontinuation in one-fourth of patients. Given that not all patients with VTE are meant to receive lifelong anticoagulants, it is possible that most patients who discontinued did so based on the discretion of their health care professional. We were able to identify clinical events preceding switching in approximately half the patients, most of which were health care encounters or diagnostic or prognostic procedures for VTE. However, we must be cautious in assigning causation to these events. With claims data, it is not possible to know whether these events were associated with these therapy changes

### Limitations

Our findings should be interpreted with study and data limitations in mind. In insurance claims, only clinical events that result in a billing transaction can be identified. If a patient discontinued treatment due to minor bleeding that was not billed, such an event was missed. It is also possible that a change in formulary, rather than a clinical event, could have been associated with switching. In our assessment of VTE risk factors, we focused on major identifiable transient risk factors^15^; it is possible that some patients with VTEs categorized as unprovoked had the event provoked by minor factors or transient factors not recorded in the data, such as air travel. Although we required that patients were VTE free for at least 365 days prior to the index VTE hospitalization, some may have had a VTE episode prior to that and thus had a history of prior VTE.

Finally, exposure to OACs was assessed based on dispensing, not actual consumption, which may lead to exposure misclassification, particularly for warfarin, especially if patients stopped or titrated the medication or purchased it through other noninsurance-related means.^27^ We also did not have information on medications given during any inpatient stay, including the index VTE hospitalization. Thus, while only approximately 35% of patients receiving warfarin had parenteral therapy before initiating warfarin recorded in the data, it is possible that a higher percentage of patients received it in the hospital.

## Conclusions

In this large cohort study of 298 609 patients with VTE, we observed a substantial shift in the use of OACs over the course of a decade, with DOACs (especially apixaban) becoming overwhelmingly preferred over warfarin. Most patients completed the initial 3 months of OAC therapy with a median treatment duration of approximately 6 months. Treatment duration was, on average, longer for patients with PE and shorter for patients with a history of bleeding. These insights into OAC use have implications when designing comparative effectiveness and safety studies of OACs for VTE recurrence.

## Notes

Supplement 1. **eTable.** *International Classification of Diseases* (*ICD*) *-9* and *-10* Diagnostic Codes for Identifying Venous Thromboembolism

Supplement 2.
Data Sharing Statement
