# Supplementary material for: Trends in the Use of Oral Anticoagulants for Adults With Venous Thromboembolism in the US, 2010-2020
Source: JAMA Netw Open. 2023 Mar 22;6(3):e234059. doi: 10.1001/jamanetworkopen.2023.4059 (PMC10034573; doi:10.1001/jamanetworkopen.2023.4059)
Supplement: Supplement 1. — eTable. International Classification of Diseases (ICD) -9 and -10 Diagnostic Codes for Identifying Venous Thromboembolism [file jamanetwopen-e234059-s001.pdf]

Supplementary Online Content

Iyer GS, Tesfaye H, Khan NF, Zakoul H, Bykov K. Trends in the use of oral anticoagulants for adults with venous thromboembolism in the US, 2010-2020. *JAMA Netw Open.* 2023;6(3):e234059. doi:10.1001/jamanetworkopen.2023.4059

eTable. *International Classification of Diseases* (*ICD*) -*9* and -*10* Diagnostic Codes for Identifying Venous Thromboembolism

This supplementary material has been provided by the authors to give readers additional information about their work.

© 2023 Iyer GS et al. *JAMA Network Open.*

eTable. *International Classification of Diseases* (*ICD*) *-9* and *-10* Diagnostic Codes for Identifying Venous Thromboembolism

For eligibility/index VTE event: presence of any of these codes in the primary position on discharge summary of an inpatient stay.

For clinical reasons for treatment modification during follow-up: presence of any of these codes in any encounter

**Pulmonary embolism**	
ICD 9 CM	415.11, 415.12, 415.13, 415.19
ICD 10 CM	I26.01, I26.02, I26.09, I26.90, I26.92, I26.99
**Deep vein thrombosis**	
ICD 9 CM	451.11, 451.19, 451.2, 451.81, 451.83, 451.84, 451.89, 451.9, 452, 453.0, 453.1, 453.2, 453.3, 453.4, 453.41, 453.42, 453.82, 453.83, 453.84, 453.85, 453.86, 453.87, 453.89, 453.9,
ICD 10 CM	I80.1, I80.20, I80.21, I80.22, I80.23, I80.29, I80.3, I80.8, I80.9, I81, I82.0, I82.1, I82.210, I82.220, I82.290, I82.3, I82.40, I82.41, I82.42, I82.43, I82.44, I82.49, I82.4Y, I82.4Z, I82.60, I82.62, I82.890, I82.90, I82.A1, I82.B1, I82.C1

© 2023 Iyer GS et al. *JAMA Network Open.*
