# Supplementary material for: Trends in the Use of Oral Anticoagulants for Adults With Venous Thromboembolism in the US, 2010-2020
Source: JAMA Netw Open. 2023 Mar 22;6(3):e234059. doi: 10.1001/jamanetworkopen.2023.4059 (PMC10034573; doi:10.1001/jamanetworkopen.2023.4059)
Supplement: Supplement 2. — Data Sharing Statement [file jamanetwopen-e234059-s002.pdf]

Data Sharing Statement

Iyer. Trends in the Use of Oral Anticoagulants for Adults With Venous Thromboembolism in the US, 2010-2020. *JAMA Netw Open*. Published March 22, 2023.
doi:10.1001/jamanetworkopen.2023.4059

Data
Data available: No

Additional Information
Explanation for why data not available: Individual patient data cannot be shared due to Data Use Agreements with data providers. Data dictionary is available upon request (kbykov@bwh.harvard.edu).
